# Successful treatment of bepridil-induced intraoperative torsades de pointes by isoproterenol infusion

**DOI:** 10.1186/s40981-021-00475-3

**Published:** 2021-10-13

**Authors:** Ken Shimano, Kyungho Chang, Yoshiki Hara, Atsushi Yasuda, Shigehito Sawamura

**Affiliations:** Department of Anesthesia and Critical Care, Teikyo University School of Medicine, 2-11-1 Kaga, Itabashi, Tokyo, 173-8606 Japan

**Keywords:** Bepridil, QT interval, Torsades de pointes, Isoproterenol, Bradycardia

## Abstract

**Background:**

Several types of antiarrhythmic drugs are known to induce QT prolongation and torsades de pointes.

**Case presentation:**

An 84-year-old man was scheduled for open gastrectomy for residual cancer. He had been prescribed bepridil for atrial fibrillation that converted to sinus rhythm with prolonged QT interval in the operating room. After the surgery was initiated under general and epidural anesthesia, the patient’s heart rate decreased to 50/min and multifocal premature ventricular contractions appeared, followed by several episodes of torsades de pointes, each lasting for 5 to 15 s. Infusion of isoproterenol was started (0.01 μg/kg/min), and the heart rate was maintained at around 80/min. Premature ventricular contractions disappeared, and torsades de pointes did not recur during the surgery. The operation was completed uneventfully. The serum bepridil concentration was found to be extremely high postoperatively.

**Conclusions:**

Bepridil-induced intraoperative episodes of torsades de pointes were successfully treated by increasing the heart rate with isoproterenol.

## Background 

Apart from congenital causes, long QT syndrome can also be secondary to several medications including antiarrhythmics known to induce QT prolongation and torsades de pointes (TdP), termed the acquired long QT syndrome. Bepridil has been shown to convert atrial fibrillation (AF) to sinus rhythm (SR) in patients with persistent AF [1, 2]. Although marked QT prolongation and TdP reportedly occur in 2.8% and 0.9% of patients on bepridil treatment, respectively [3], there have been few reports describing intraoperative TdP induced by preoperative bepridil therapy.

## Case presentation

Written informed consent was obtained from the patient for the publication of this case report and accompanying images.

**Fig. 1:**
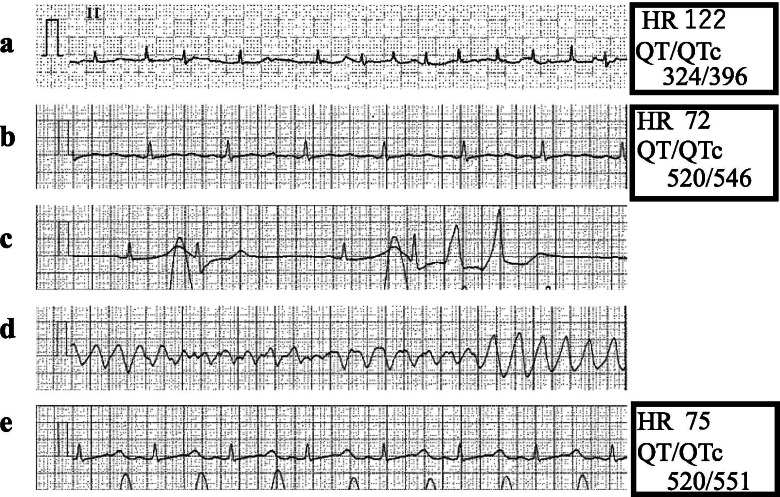
Perioperative electrocardiographic changes. **a** One month before the operation: atrial fibrillation. **b** On admission to the operating room: sinus rhythm. **c** Eighteen min after the start of surgery: short runs of multifocal premature ventricular contractions (PVCs) are observed. **d** Twenty min after the start of surgery: torsades de pointes (TdP) lasting for 15 s. **e** After the start of isoproterenol: sinus rhythm: PVCs and TdP have disappeared

An 84-year-old male was scheduled for open gastrectomy for residual cancer. He was prescribed with bepridil (200 mg/day) and edoxaban (60 mg/day) for AF. Electrocardiography (ECG) one month before the operation revealed AF with a heart rate (HR) of 122/min and QT/QTc interval of 324/396 ms (QTc: QT corrected with the Framingham formula, Fig. 1a). Echocardiography revealed an ejection fraction of 57% without wall motion abnormalities. He had no history of syncope. His renal and hepatic functions were normal. Edoxaban was withheld for 2 days before the surgery, while bepridil was continued until the morning of surgery to maintain control of HR.

On admission to the operating room, ECG revealed normal SR with a HR of 72/min and QT/QTc interval of 520/546 ms (Fig. 1b). His blood pressure (BP) was 127/66 mmHg. A thoracic epidural catheter was inserted via the T7/T8 interspace. General anesthesia was induced with fentanyl 100 μg, propofol 80 mg, and rocuronium 50 mg and maintained with remifentanil 0.1 μg/kg/min, sevoflurane 1.5%, and rocuronium as required. Ropivacaine (0.2%, 7 ml) was administered through the epidural catheter. After the administration of general and epidural anesthesia, his HR and BP gradually decreased (50/min and 90/40 mmHg, respectively). Eighteen minutes after the start of surgery, multifocal premature ventricular contractions (PVCs) and short runs were observed (Fig. 1c). Then, several episodes of TdP, each lasting for 5 to 15 s, occurred and terminated spontaneously (Fig. 1d). His systolic BP decreased to 30 mmHg, and pulse waves were not detected during TdP. The serum potassium level at this time was 3.6 mEq/L.

Isoproterenol (0.01 μg/kg/min) was started to increase the HR and counteract the QT-prolonging effect of bradycardia. A few minutes after the start of isoproterenol, once the HR increased to 80/min, PVCs, short runs, and TdP disappeared and were not observed thereafter (Fig. 1e). Subsequently, potassium 10 mEq and magnesium 20 mEq were administered slowly.

The surgery was continued and completed as planned. The operation time was 4 h and 26 min, and the blood loss was 130 ml. The patient was extubated uneventfully and admitted to the intensive care unit (ICU) with continued isoproterenol infusion. No life-threatening arrhythmias were observed in the ICU, though QT prolongation persisted.

The next morning, SR converted to AF with a HR of 120/min and QT/QTc interval of 235/333 ms. Isoproterenol was discontinued, and carvedilol (5 mg/day) was started to control the HR. Four days later, AF again converted to SR with a HR of 89/min and QT/QTc interval of 440/538 ms. No episodes of TdP were observed until discharge from the hospital.

Bepridil was not administered postoperatively. The serum concentrations of bepridil on the second and third postoperative days (2341 and 2415 ng/mL, respectively) were found to be remarkably high with respect to the reported therapeutic range (250–800 ng/mL) [4].

## Discussion

We report a case of intraoperative QT prolongation and TdP in a patient on bepridil. TdP was successfully treated by increasing the HR with isoproterenol.

Several medications, including antiarrhythmics, are known to induce QT prolongation and TdP, a rare but life-threatening arrhythmia. Johnston et al. analyzed 46 cases of perioperative TdP in the literature between 1978 and 2011 [5]. The main preceding events were QT prolonging drugs (30%), hypokalemia (26%), and bradycardia (15%). The mean QTc at the time of TdP was 575 ms.

Bepridil is indicated for conversion of AF to SR in patients with persistent AF [1, 2]. However, bepridil reportedly also induces QT prolongation and TdP because of its multiple ion-channel blocking action, including that of potassium channels [3]. Careful follow-up is therefore highly recommended for the prevention of TdP during its use, especially in elderly patients. The risk factors for TdP in patients treated with bepridil include QT prolongation, bradycardia, hypokalemia, and advanced age [3].

QT/QTc interval on admission to the operating room was prolonged (520/570 ms) in our patient, though that measured 1 month before surgery was within normal limits (324/396 ms). It is known that QT/QTc intervals can fluctuate widely depending on the patient’s activity, posture, circadian rhythm, and food intake. Therefore, we should keep in mind that preoperative assessment of QT intervals cannot always predict the intraoperative occurrence of TdP [6].

Conversion of AF to SR was related to QT prolongation in our patient. With the conversion of AF to SR, the QT/QTc changed from 324/396 ms to 520/546 ms preoperatively and from 235/333 ms to 440/538 ms postoperatively. It is not clear whether QT prolongation was caused by rhythm conversion itself or by decreased HR. There is one report describing TdP episodes soon after the conversion of AF to SR in patient on bepridil therapy [7].

Bradycardia is known to exacerbate QT/QTc prolongation and eventually trigger TdP in acquired long QT syndrome. During intraoperative management, opioids used for general anesthesia (fentanyl and remifentanil) and the sympathetic blocking properties of thoracic epidural anesthesia can often induce bradycardia. In fact, the HR decreased from 72 to 50/min after the induction of general and epidural anesthesia in our patient.

We should also pay attention to electrolyte abnormalities, especially hypokalemia and hypomagnesemia in patients with long QT. Hypokalemia (3.6 mEq/L) could have contributed to the occurrence of short runs and TdP in our patient, though they disappeared immediately after the start of isoproterenol infusion, that is, before the correction of hypokalemia.

Advanced age is reportedly associated with QT prolongation. Furthermore, sevoflurane reportedly causes greater QTc interval prolongation in elderly patients than in younger patients [8]. However, the extent to which sevoflurane increases the risk of life-threatening arrhythmias in patients with acquired long QT syndrome remains unclear.

Assessment of serum concentration of bepridil can be useful to predict the occurrence of TdP, because the QTc interval is reportedly significantly associated with serum bepridil concentration [4]. In our patient, serum bepridil concentrations measured on the second and third postoperative days were remarkably high with respect to the reported therapeutic range, suggesting that intraoperative QT prolongation and TdP were induced by bepridil.

Thus, preoperative evaluation of the risk of life-threatening arrhythmias is crucial in patients on QT interval-prolonging drugs. Preoperative withdrawal of bepridil should have been considered in our case, though its serum concentration reportedly persists for several weeks after cessation [6]; the serum concentration of our patient did not change for one day (2341 vs. 2415 ng/mL).

As bradycardia triggers QT prolongation and TdP in acquired long QT syndrome, increasing the HR could be a rational treatment strategy. In our patient, increasing HR pharmacologically with isoproterenol showed immediate and remarkable effects in the treatment of bepridil-induced TdP, as has been previously reported in an ICU patient [6]. As isoproterenol can be started quickly, we think it is worth trying in the operating room. Cyclic AMP amplification by isoproterenol can activate potassium channels and counteract QT prolongation caused by bepridil. However, the QT interval did not change after isoproterenol infusion in our patient. Temporary pacing could also have been effective in increasing the HR, but we did not try it because percutaneous pacing can often fail and the preparation for trans-jugular pacing can be time-consuming.

Lastly, although we chose isoproterenol treatment to increase the HR and counteract the QT-prolonging effect of bradycardia, bolus administration of magnesium could also be considered as an immediate treatment for TdP.

## Conclusions

QT prolongation and repeated episodes of TdP were observed during combined general and epidural anesthesia in a patient on bepridil therapy. TdP was successfully treated by pharmacologically increasing the HR with isoproterenol infusion.

## Data Availability

Not applicable

## Abbreviations

TdP: Torsades de pointes
AF: Atrial fibrillation
SR: Sinus rhythm
ECG: Electrocardiography
HR: Heart rate
BP: Blood pressure
PVC: Premature ventricular contraction
ICU: Intensive care unit
